# Retraction Note: HMGB1 Facilitated Macrophage Reprogramming towards a Proinflammatory M1-like Phenotype in Experimental Autoimmune Myocarditis Development

**DOI:** 10.1038/s41598-022-10210-2

**Published:** 2022-04-08

**Authors:** Zhaoliang Su, Pan Zhang, Ying Yu, Hongxiang Lu, Yanfang Liu, Ping Ni, Xiaolian Su, Dan Wang, Yueqin Liu, Jia Wang, Huiling Shen, Wenlin Xu, Huaxi Xu

**Affiliations:** 1 Department of Immunology, Jiangsu University, Zhenjiang, 212013 China; 2 The Central Laboratory, the Fourth Affiliated Hospital of Jiangsu University, Zhenjiang, 212001 China

Retraction of: *Scientific Reports* https://doi.org/10.1038/srep21884, published online 22 February 2016

The Authors have retracted this Article.

After publication, the Authors informed the journal that Fig. 4 contained incorrectly reported results, and the images in Fig. 5 had been published elsewhere. In addition, concerns were raised regarding partial overlap in the flow cytometry plots in Fig. 2F (HMGB1 and HMGB1 + anti-TLR4 groups). The Authors are unable to provide raw data used to produce the figures, and therefore retract this Article.

All Authors agree with this retraction.

